# Structure and Flame-Retardant Actions of Rigid Polyurethane Foams with Expandable Graphite

**DOI:** 10.3390/polym11040686

**Published:** 2019-04-16

**Authors:** Yongjun Chen, Yuanfang Luo, Xiaohui Guo, Lijuan Chen, Tiwen Xu, Demin Jia

**Affiliations:** 1 Key Lab of Guangdong High Property and Functional Macromolecular Materials, Department of Polymer Materials and Engineering, South China University of Technology, Guangzhou 510640, China; yjchen@scut.edu.cn (Y.C.); psyfluo@scut.edu.cn (Y.L.); 15602325346@163.com (X.G.); psdmjia@scut.edu.cn (D.J.); 2 Guangdong Provincial Key Laboratory of Functional Soft Condensed Matter, Department of Polymeric Material and Engineering, School of Materials and Energy, Guangdong University of Technology, Guangzhou 510006, China

**Keywords:** expandable graphite, rigid polyurethane foams, structure, flame retardancy, representation

## Abstract

In this paper, rigid polyurethane foams that were filled with expandable graphite (RPUF/EG) composites were prepared by the liquid blending method, and then the structure and flame retardancy performance of materials were investigated through optical microscope, scanning electron microscope, limit oxygen index, cone calorimeter, thermogravimetric analysis coupled to fourier transform infrared spectrum, and X-ray photoelectron spectroscopy. The results showed that a large number of EG could be good to the exhibition of flame retardancy of RPUF, where the optimal material was found at loading 15 phr EG that showed an increased limit oxygen index value and a decreased calorific or fuming value. TGA coupled FTIR and XPS revealed that EG could disassembled before RPUF under heating treatment, and it could form a pyknotic and enahnced residual carbon layer on RPUF surface after the fire, which restricted the transfer of gas, like oxygen or heat into PU matrix, finally resulting in the promotion of flame retardancy of RPUF.

## 1. Introduction

The rigid polyurethane foams (RPUF) is a kind of foamed plastics, and it could obtain a permanent deformation until a critical force is conducted [1]. RPUF owns some virtues, like light weight, high compressive strength, optimal obstructing performance, favourable adhesive property, and benign processing property, and it is used widely in architecture, automobile, electronics, packaging, shipbuilding, aerospace, and so on [2,3,4]. With large consumption in the economy, some defects of RPUF also show up in using, which seriously confuse people for the free-burning, large total smoke release, and quick rate of firing that immensely limit the application of RPUF [5].

When considering the situation, researchers conducted lots of investigations regarding how to promote RPUF’s the flame retardancy performance. In general, two methods were mainly adopted [6,7,8,9]. One way was adding the non-reactive flame retardant [10], which was confirmed to be a simple and effective approach to improve the flame resistance. Wang et al. used a phosphorous-nitrogen intumescent flame-retardant, 2,2-diethyl-1,3- propanediol phosphoryl melamine (DPPM) to improve the flame retardancy of RPUF, where the limit oxygen index (LOI) value was increased to 29.5% when loading 25 phr DPPM [11]. Another way was found to introduce the fire retardant elements or heat resistant groups into the RPUF molecules, which could be observed in the report [12]. Although the latter one could endow an excellent flame retardancy of material, the processing or price of modified RPUF were complicated and expensive, which limits the development of this method.

For the moment, the additive flame retardants, such as magnesium hydroxide [13], aluminum hydroxide [14], red phosphorus [15], nitrogen flame retardant, and intumescent flame retardants [16,17] were widely used in RPUF. Expanded graphite (EG) is a loose and vermiculate material that was obtained from the intercalation, puffing on natural graphite, and it could promote the flame retardancy of polymers [18,19,20,21]. Khalili demonstrated that EG incorporated with natural fiber could improve the fire resistivity of epoxy, especially at 5 wt % loading of EG, and that the material obtained a sufficient self-extinguishing property [22]. Gama used castor oil and EG to fill polyurethane foams (PUFs), and the results demonstrated an increase of thermal and electrical conductivities by loading EG from 0.5% to 1.5% (*w*/*w*) [23]. In this paper, the RPUF/expandable graphite (RPUF/EG) composites were prepared by adding EG into PU matrix before foaming. The foam microstructure, thermal stability, degradation mechanism, flame retardancy performance, and retardant mechanism were illustrated, which is good for the further application of RPUF.

## 2. Experimental

### 2.1. Materials

**Scheme 1:**
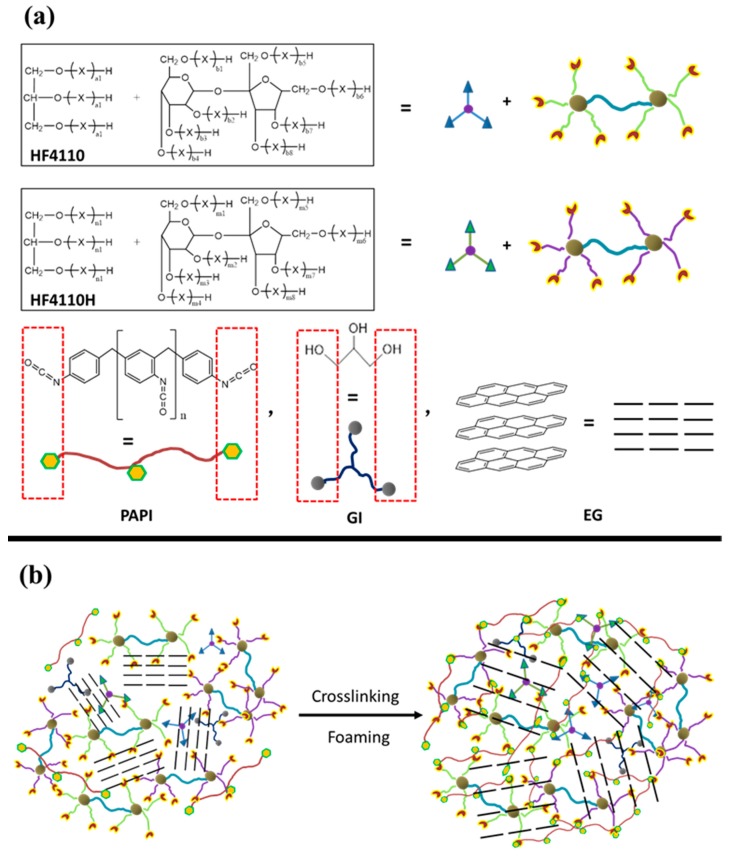
(**a**) Chemical structures of the main components of rigid polyurethane foams with expandable graphite and (**b**) illustration of the crosslinking and foaming processes of the RPUF/EG composites.

Polyether polyol for rigid foam (HF-4110H, HF-4110) were purchased from Hengfeng polyurethane industrial co. LTD (Shaoxing, China). The primary properties of HF-4110H and HF-4110 were as follows: hydroxyl value, both 430 ± 30 mg KOH equiv/g; viscosity (25 °C), 1500–2000, 4500–6500 mPa·s, respectively. Polyphenylpolymethylene isocyanate (PAPI) was provided by Hebi chemical co. LTD (Shanghai, China). The primary physical indices were as follows: –NCO weight percent, 31%; viscosity (25 °C), 200 mPa·s; degree of functionality was 2.7; and, expandable graphite (EG) (ADT 249) was supplied by Shijiazhuang aidi kepeng flame retardant materials co. LTD (Shijiazhuang, China) with an expansion rate of 250 ml/g, and their chemical structures are displayed in Scheme 1a; 33 wt % triethylene diamine dipropylene glycol solution (A33) was purchased from Hongpu chemical technology co. LTD (Shanghai, China); silicone oil (AK-8805), a kind of silicon-carbon bond non-hydrolyzable polysiloxane-polyether copolymer, as the foam stabilizer was obtained from Liangkun chemical co. LTD (Guangzhou, China); glycerinum (GI) was of AR grade provided by Guangzhou chemical reagent factory; deionized water (H_2_O) was self-made. 

### 2.2. Preparation of Rigid Polyurethane Foams (RPUF) with Expandable Graphite

The applicable components of HF-4110H, HF-4110, A33, AK-8805, GI, H_2_O, and EG were rapidly mixed together via an SFJ-400 high speed dispersing machine (Shanghai, China) at 500 rpm in a 1000 mL plastic cup. Subsequently, PAPI was added into the mixture with a continued stirring for 5 min, after which the compounds were injected into the mould (300 mm × 300 mm × 100 mm) for foaming or molding when the bubbles escaped out. The demoulding procedure was done after 10 min treatment, followed by a curing process at 80 °C for 5 h. Finally, the prepared samples were taken to meet tests. The formulas of RPUF/EG composites that are listed in Table 1 and Scheme 1b show the crosslinking and foaming processes.

### 2.3. Characterization

A BX51 Polarizing optical microscope (Olympus Company, Tokyo, Japan) and a 1530VP Scanning electron microscopy (SEM) observed the foam structures of the RPUF/EG composites (LEO Company, Berlin, Germany), respectively. 

The Limit Oxygen Index (LOI) values were obtained using the Fire Testing Technology (FTT, East Grinstead, UK) Dynisco LOI instrument according to ISO 4589-2:1996, and the size was 120 mm × 6 mm × 3 mm. The LOI measurements for each specimen were conducted five times. 

The cone colorimeter tests were done using a cone calorimeter (Fire Testing Company, UK) at a heat flux of 35 kW/m^2^ with specimen dimensions of 100 × 100 × 25 mm^3^ according to ISO 5660 standards.

The thermal stability of composite under nitrogen atmosphere was investigated via a NETZSCH 209 thermogravimetric analyer (TGA, Munich, Germany) with the temperature range from 25 to 800 °C at 10 °C/min. To trace the typical quenching fragments of composite, the thermogravimetry coupled to infrared spectrum analysis was employed with a Perkin-Elmer STA6000 simultaneous thermal analyzer and a Perkin-Elmer FRONTIER fourier transform infrared spectrometer (FTIR, Waltham, MA, USA). The nitrogen was utilized as carrier gas for the volatile products at flow rate of 50 ml/min, and the temperature was scanned from 40 to 800 °C at a heating rate of 20 °C/min.

The macro morphology of residues from cone calorimeter was observed with a EOS400D digital camera (Canon, Tokyo, Japan). Simultaneously, the elemental compositions of residues were analyzed via a PHI QUANTERA-II SXM X-ray photoelectron spectrometer (XPS) (ULVAC-PHI, Chigasaki, Japan) with Al Kα radial (1486.6 eV). The data were adjusted according to C1s bonding energy at 284.8 eV with Multipak 2.2 Software.

## 3. Results and Discussion

### 3.1. Microstructures of Foam

**Figure 1:**
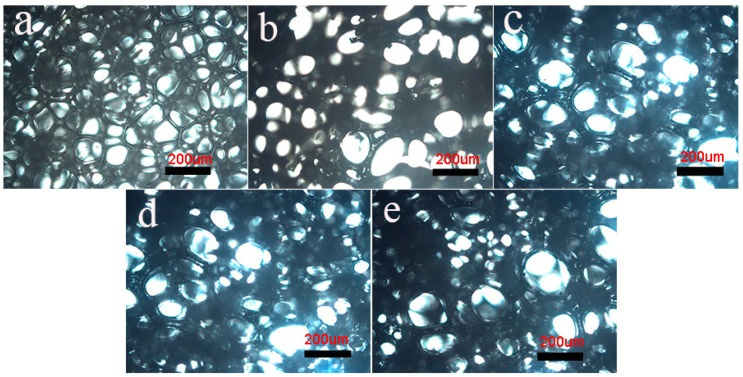
Polarizing microscope photos of the pure RPUF and RPUF/EG composites: (**a**) RPUF, (**b**) RPUF/EG5, (**c**) RPUF/EG10, (**d**) RPUF/EG15, and (**e**) RPUF/EG20.

Figure 1 illustrates the microstructures of RPUF/EG foams by optical microscope. As seen, some anomalous polygons with different dimensions are observed. In Figure 1a, the uniform foams exhibit a smaller average aperture when compared to others. Via adding EG, the increased average aperture and decreased number of foams in unit area are obtained, indicating an appearance of combined bubbles, which is enhanced via increasing EG. In Figure 1c,d, some obvious wrinkles show up around bubbles, reflecting the decrease of compactness and intensity for foams. 

**Figure 2:**
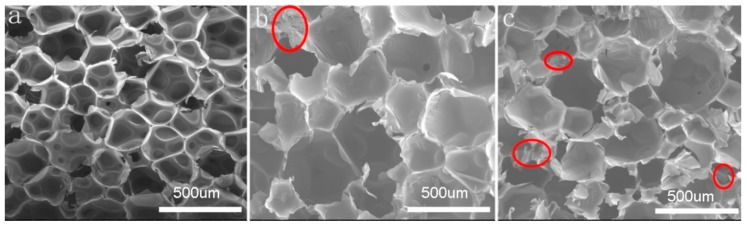
Scanning electron microscopy (SEM) photos of the pure RPUF and RPUF/EG composites: (**a**) RPUF, (**b**) RPUF/EG10, and (**c**) RPUF/EG20.

Further, Figure 2 displays the SEM morphology of RPUF/EG foam. It can be seen that the average size and heterogeneity of foams are increased by adding EG into RPUF matrix, especially at higher EG loading, which is in line with the results from Figure 1. Simultaneously, with the increase of EG, the number of cracked cells is increased for the traverse and adsorption of EG (signed with circles) onto the margin of bubbles, which could form an isolated layer [24]. 

### 3.2. Compression Strength

**Figure 3:**
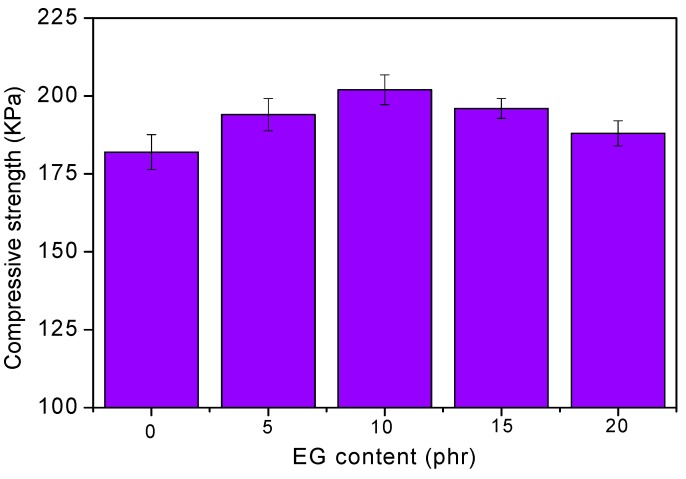
Effect of EG content on the compressive strength of RPUF.

Figure 3 shows the compression strengths of RPUF/EG. As seen, the strength of material is increased via increasing EG until a threshold value is obtained at 10 phr EG. The compression strength of RPUF is determined by the uniformity of bubbles, where the more the uniformity is, the better the strength will be. As seen from Figure 2, the bubble structures of RPUF could be integrity and uniformity when loading EG at lesser contents, where EG could also play as a reinforcing material that leads to the promotion of compression strength. When loading much more EG, its self-agglomeration would change the homogeneity of viscosity, resulting in a destruction of the bubble structure. Subsequently, the compression strength of RPUF was decreased.

### 3.3. LOI Tests

**Figure 4:**
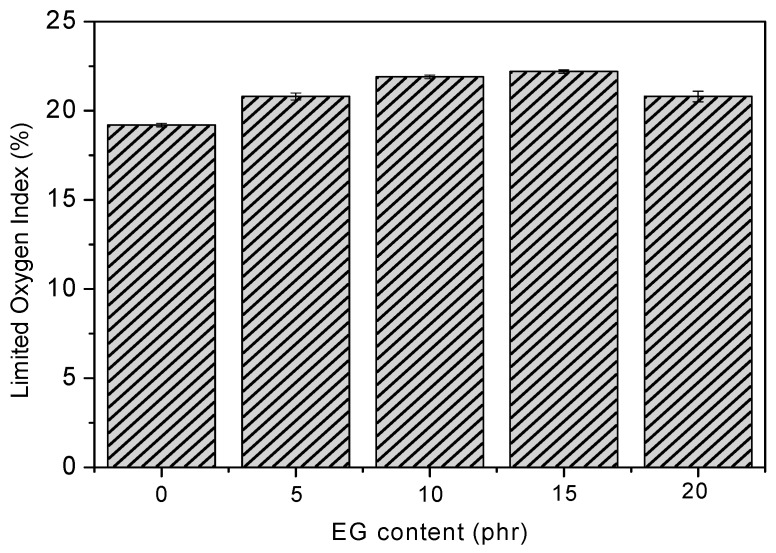
Effect of EG on the limit oxygen index (LOI) values of RPUF.

The flame-retardant behavior of RPUF/EG was first investigated using LOI tests, and the results are shown in Figure 4. It can be viewed that LOI of RPUF is increased via increasing the EG until a threshold value is obtained at 15 phr EG, where the LOI is 22.2% when compared to 19.2% of pure RPUF. EG could expand to be carbon layer and adsorb onto the surface of the materials [19,25], which stops the migration of air or heat. All of the samples after adding EG display the promotions of LOI value to be 20.8%, 21.9%, 22.2%, and 20.8%, respectively, implying the positive effect of EG on the flame-retardant performance of RPUF. The positive effect of EG itself on promoting LOI is also impacted by the dispersion of EG filler in RPUF matrix, where lots of EG with bad dispersion might decrease the LOI value. On the other hand, the changes of LOI are still small after adding EG, because of the poor adhesion between RPUF and EG [26].

### 3.4. Cone Calorimeter

Cone calorimeter was finished to further assess the flame retardancy of RPUF, and Table 2 lists partial data. The time to ignition (TTI) is increased via adding EG, and the peak value of heat release rate (PHRR), total heat release (THR), total smoke release (TSR) are all decreased with the increase of EG, all of which indicate the enhanced flame retardancy of RPUF/EG composites. This phenomenon should be due to the effective assimilation on heat by EG that delayed the burning of foams.

**Figure 5:**
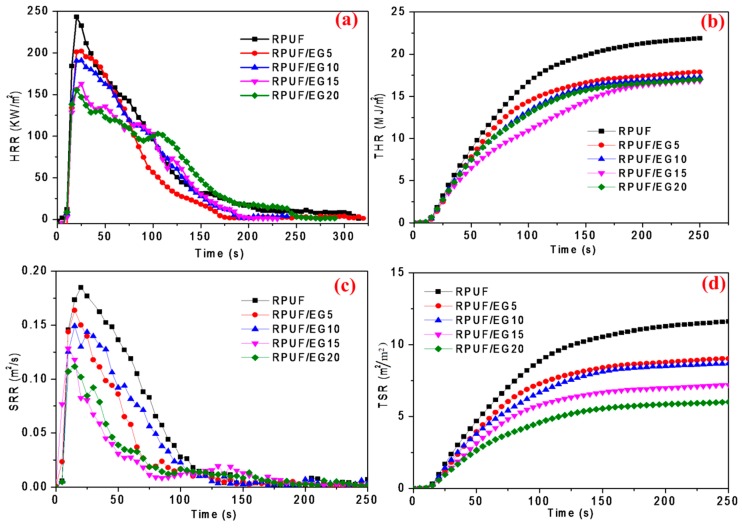
(**a**) Heat release rate (HRR), (**b**) total heat release (THR), (**c**) SRR, and (**d**) total smoke release (TSR) curves of RPUF filled with different EG.

Furthermore, Figure 5 illustrates the time depending curves of burning indexes. The heat release rate (HRR) is an important value in evaluating the fire risk. As seen in Figure 5a, HRR is significantly decreased by increasing EG at the initial burning for 70 seconds, where the PHRR is diminished by 36.2% at 20 phr EG loading. In Figure 5b, the total heat release (THR) is also obviously diminished after burning for the initial 50 seconds, and the smallest value is obtained at 15 phr EG. This can be interpreted that the burning of EG would happen ahead of foams, whereby the reaction might absorb a lot of heat, leading to the decline of HRR and THR values. It is well known that smoke is the most dangerous cause of death during the fire, thus the control of smoke release rate (SRR) is critical in judging the flame retardancy of material. Figure 5c,d illustrate the SRR and TSR curves of the RPUF/EG composites. As seen in Figure 5c, the rate reaches the top point in 20 seconds after burning. When comparing to pure RPUF, the peak is lowered after adding EG, where the peak value is decreased by 11.4%, 19.5%, 30.8%, and 40.0% at 5 phr, 10 phr, 15 phr, and 20 phr, respectively. Simultaneously, increasing EG decreases TSR, and when increasing EG, the final stable TSR is severally reduced by 22.4%, 24.1%, 37.9%, and 48.3%. Therefore, EG plays a positive role in restraining the generation of smoke for RPUF. Above all, the addition of EG into RPUF could significantly improve the flame retardancy performance of RPUF.

### 3.5. TGA-FTIR Analysis of RPUF/EG Samples

The influence of EG on the thermal stability of RPUF was conducted and Table 3 lists the data. It can be viewed that all of the foams exhibit three degradation stages under heating, which are distinguished to be low temperature region (100~260 °C), medium temperature area (260~390 °C), and high temperature zone (390~600 °C) that are consistent with other report [27]. Basing on the data of low temperature section, the temperature values at 5% and 10% mass loss are both decreased via increasing EG, also with the integral decline for temperature at 50% mass loss in the medium temperature area. This depends on the low expansion temperature of EG (165 °C), which leads to the formation of a carbon layer on the polymer surface before PU decomposition, thus the polymer was protected and its thermal stability was also improved. However, there are no apparent changes about the residual percentage of RPUF when increasing EG.

**Figure 6:**
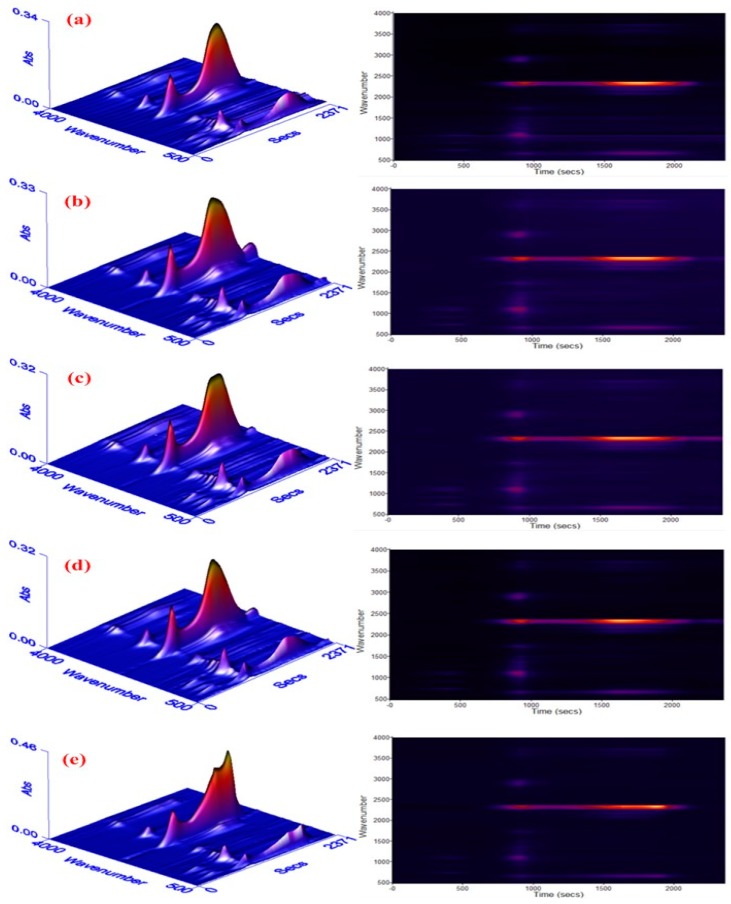
Three-dimensional fourier transform infrared spectrometer (3D FTIR) spectra of escaped gases from the degradation of RPUF: (**a**) pure RPUF, (**b**) 5phr EG, (**c**) 10 phr EG, (**d**) 15 phr EG, and (**e**) 20 phr EG.

To clearly view the thermo-decomposition process, the escaped gases during degradation of materials were analyzed via TGA coupled to FTIR, and the results with three-dimensional (3D) spectra are performed in Figure 6. Different colours reflect different intensities of absorption peaks, where the yellow, red and blue show a decline tendency of intensity. 

As seen, three absorption areas show up for all composites. With the first section below 210 °C, there are peaks around 1000 cm^−1^ for pure RPUF, such as 1120 cm^−1^ (C–O–C peak), 722 cm^−1^ (HCN peak) [26]. This indicates that the fractional C-O bonds in RPUF had broken up, resulting in the birth of small molecules. After adding EG, the broken of partial C-O bonds also appears, and it becomes more serious with the increase of EG until a threshold value is obtained at 10 phr EG loading. When loading 20 phr EG, the peak intensities are lower than that of pure RPUF, indicating an effective restraint of polymer degradation. This can be explained by the compactness of RPUF being potentially destroyed because of the poor consistency between PU and EG when loading less than 10 phr EG. Meanwhile, insufficient EG can not form a stable carbon layer adsorbing on polymer surface to stop heat from going inside. Thus, this leads to a reduced thermal stability of polymer materials. Via continuously increasing EG, a compact and steady carbon layer could be produced on a PU surface to quarantine heat or gas flow in the end. This conquers the negative impact of compatibility, inducing a promoted thermal stability of materials. In the second degradation stage, some new peaks about C–H (around 2930 cm^−1^), C–O of ester (1257cm^−1^), C–N of amine (1220~1320 cm^−1^), and C=O (1744 cm^−1^) arise, implying a worse decomposition reaction of PU. Simultaneously, the peak variations around 2300 cm^−1^ standing for the vibration of –NCO, CO_2_ exhibit a similar trend when comparing to that of the low temperature region when increasing EG, which is also caused by the competition between the poor compatibility of PU/EG and carbon layer’s formation. The degradation of polymer becomes more serious when the temperature exceeds 540 °C. According to the peak heights in 3D pictures, the gas release is obviously decreased at 20 phr EG loading when compared to pure RPUF, which implies an improved thermal stability of RPUF/EG-20.

### 3.6. Macro Morphology of Residues from Cone Calorimeter

**Figure 7:**
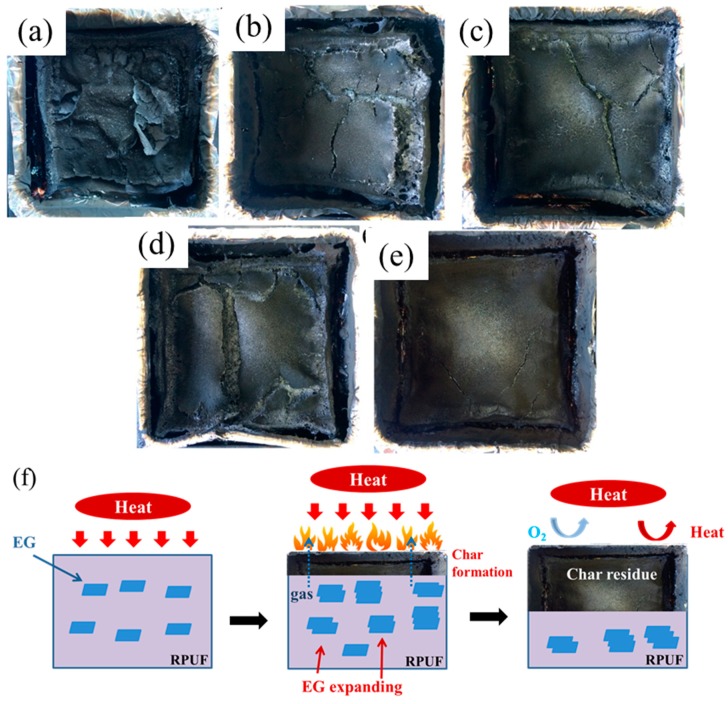
Digital photographs of the residue char after the cone calorimeter test: (**a**) RPUF, (**b**) RPUF/EG5, (**c**) RPUF/EG10, (**d**) RPUF/EG15, (**e**) RPUF/EG20, and (**f**) a schematic illustration of the flame retardant and restraining the heat or gas exchange mechanism of EG reinforced RPUF composites.

Figure 7a–e depicts the influence of EG on the macro morphology of residues from cone calorimeter. As seen, the residual carbon layer of pure RPUF is slight, and it would be easily broken, resulting in a limited barrier action to heat or gas, which means a bad performance of the flame retardancy of material. With the increase of EG, an integrated carbon layer becomes unambiguous, even though the cracks still exist, which indicates an improved barrier property of residual carbon layer. Thus, an abundant EG could form an unabridged and stable residual carbon layer on PU surface after burning, restraining the heat or gas exchange from the inside to outside, and then the flame retardancy of RPUF is promoted. The significant improvement on the flame retardant and restraining the heat or gas exchange mechanism of EG reinforced RPUF composites is explained by suggesting a flame retardancy mechanism in Figure 7f. Graphite expansion develops the worm-like and exfoliated structures of the EGs, which improves the barrier property of the residual carbon layer. Meanwhile, the flame and restrain mass transfer from RPUF to the heat source has been suffocated, which can prevent further decomposition of the inner material.

### 3.7. XPS Analysis of Residues from Cone Calorimeter

**Figure 8:**
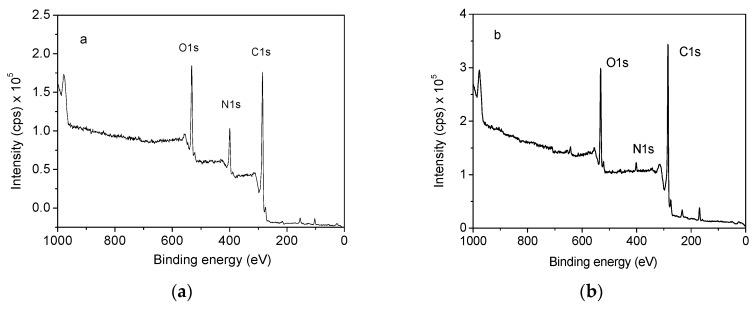
X-ray photoelectron spectrometer (XPS) spectra of (**a**) RPUF and (**b**) RPUF/EG20.

To further determine the joint charring effect from RPUF/EG system, the elemental compositions in residues from the burning of RPUF and RPUF/EG20 were all detected using XPS. Figure 8 illustrates the total spectra of the RPUF and RPUF/EG20 composite. As seen, the relative intensity of C1s/N1s is obviously increased when adding EG20 into RPUF matrix, which means the successful adsorption of EG onto RPUF surface that induced the change of chemical environment around nitrogen.

**Figure 9:**
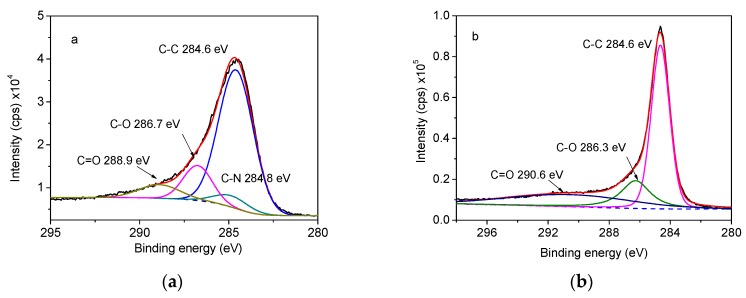
High resolution C1s XPS spectra of (**a**) RPUF and (**b**) RPUF/EG20.

Furthermore, the peak fitting program of C 1s was adopted and Figure 9 display the spectra. It can be seen that the peaks at 284.6, 284.8, 286.7, and 288.9 eV, implying the existence of C–C, C–N, C–O, and C=O bonds, respectively. After adding EG20, the peaks of C–O, C=O shift to 286.3 and 290.6 eV separately, as well as the disappearance of C–N peak, which indicates the thick coverage of EG on RPUF surface that disturbed the detection of C–N in RPUF. Simultaneously, the peak intensity data in Table 4 also demonstrate some valuable information. As seen, the relative content of C–C group reflected by the covering area changes from 49.1% to 51.4% before and after adding EG20 into RPUF matrix, also with the decrease of C–O group and the relative increase of C=O group. These all disclose an enhanced heat resistance of RPUF/EG20 material. 

Above all, EG was triumphantly absorbed onto the surface of RPUF, and some might insert into the polymer matrix. These EG could expand to be an insulating layer under heating treatment, then enhancing the compactness and strength of carbon layer on the material surface that could effectively restrict the transfer of heat and oxygen. Finally, the optimal flame retardancy of RPUF was obtained.

## 4. Conclusions

In summary, the impacts of EG on the structure and flame retardancy performance of RPUF were investigated. The results demonstrated that a large number of EG could restrict the apertures of foams in an uneven manner due to the different viscosities of polymer that are caused by the agglomeration of EG itself. Simultaneously, the thermal stability of RPUF/EG composite was decreased because of the decomposition of EG in advance at 165 °C. Fortunately, the HRR, THR, SRR, and TSR values of RPUF were obviously decreased after adding EG, especially at high EG loading. Additionally, adding EG increased the LOI and TTI values and their maximum values were obtained around 15 phr EG loading. These facts indicated the promotion of RPUF on the flame retardancy via adding EG. Through the analysis of residual carbon, appropriate EG or at large loading content could form a pyknotic and enhanced residual carbon layer that restrained the transfer of heat and oxygen into PU matrix, which induced the improvement of the flame retardancy of RPUF.

## Figures and Tables

**Table 1:** Formulas of rigid polyurethane foams/expandable graphite (RPUF/EG) composites.

Sample	RPUF (phr)	RPUF/EG5 (phr)	RPUF/EG10 (phr)	RPUF/EG15 (phr)	RPUF/EG20 (phr)
HF-4110H	70	70	70	70	70
HF-4110	30	30	30	30	30
H_2_O	3	3	3	3	3
AK-8805	2	2	2	2	2
A33	2	2	2	2	2
GI	1.0	1.0	1.0	1.0	1.0
PAPI	138	138	138	138	138
EG	0	5	10	15	20

**Table 2:** Cone calorimetric data for the RPUF filled with different EG.

Samples	TTI (s)	PHRR (kW/m^2^)	THR (MJ/m^2^)	TSR (m^2^/m^2^)
RPUF	4	243	21.8	11.6
RPUF/EG5	5	202	17.8	9.0
RPUF/EG10	6	190	17.3	8.8
RPUF/EG15	6	163	16.8	7.2
RPUF/EG20	5	155	17.0	6.0

**Table 3:** Thermogravimetric (TG) parameters of RPUF/EG composites.

Property	RPUF	RPUF/EG5	RPUF/EG10	RPUF/EG15	RPUF/EG20
temperature at 5% mass loss/°C	196.5	182.3	173.8	167.3	160.6
temperature at 10% mass loss/°C	249.8	249.8	236.3	227.3	215.6
temperature at 50% mass loss/°C	364.8	347.3	353.8	354.8	350.6
onset degradation temperature/°C	180.0	163.6	155.4	145.0	139.2
temperature at maximum mass loss rate/°C	356.5	340.0	342.5	345.0	341.8
residual percentage/%	17.5	18.2	17.3	16.7	16.6

**Table 4:** C1s fitting group content of RPUF and RPUF/EG20.

Identification	RPUF		RPUF/EG20	
	BE(eV)	A(%)	BE(eV)	A(%)
**C-C**	284.6	49.1	284.6	51.4
**C-N**	284.8	6.4	—	—
**C-O**	286.7	31.9	286.2	12.4
**C(O)O**	288.9	13.6	289.6	36.2
